# Physicians' Perspective of Telemedicine Regulating Guidelines and Ethical Aspects: A Saudi Experience

**DOI:** 10.1155/2022/5068998

**Published:** 2022-10-12

**Authors:** Dalia Yahia M. El Kheir, Sara S. Alnufaili, Raghad M. Alsaffar, Majd A. Assad, Zahra Z. Alkhalifah

**Affiliations:** ^1^Department of Family and Community Medicine, College of Medicine, Imam Abdulrahman Bin Faisal University, Dammam, Saudi Arabia; ^2^Department of Neurosurgery, King Saud Medical City, Riyadh, Saudi Arabia

## Abstract

**Methods:**

This was an observational cross-sectional study conducted among physicians working at the College of Medicine, Imam Abdulrahman Bin Faisal University (IAU), and its affiliated academic tertiary hospital, King Fahad Hospital of the University (KFHU), Eastern province-KSA. Data were collected between August 2019 and March 2020 via a structured, pretested, self-administered questionnaire distributed to 220 eligible physicians at KFHU. A final sample of 101 physicians completed our survey. Collected data was coded and analysed using SPSS, and the results presented as frequencies, percentages, and summary statistics.

**Results:**

Among our respondents, 62 (61.4%) were males, 46 (45.5%) were in the age group of 35 to 44 years, and 62 (61.3%) were Saudi. Two-thirds of physicians (58 (57.4%)) use smart devices in healthcare delivery, and 51 (50.5%) are satisfied with this use. A minority (21 (20.8%)) knew about telemedicine guidelines, 8 (7.9%) had encountered legal issues related to telemedicine use, and 52 (51.5%) were doubtful about patients' readiness for virtual care. Regarding physicians' awareness of the ethical aspects regulating the use of social media and medical apps in patient care, only 44 (45.3%) were aware of the proper reporting system if they found accounts sharing unreliable information. Nevertheless, the majority (91 (92.9%)) agreed it is essential for healthcare providers to report such accounts. Concerning physicians' awareness of the rules and regulations of online self-promotion, the majority of our respondents were unsure or unaware of such regulations (46 (45.6%) and 18 (17.8%)), respectively. Nonetheless, 67 (66.3%) of the physicians would not pay SM influencers to advertise for them.

**Conclusion:**

Two-thirds of our physicians use smart devices in healthcare delivery, with 1 in 13 having encountered related legal issues. Nonetheless, only a minority was aware of telemedicine use regulating guidelines, including physicians' online self-promotion regulations. These results highlight the necessity of targeted physicians' training on telemedicine use related guidelines, thereby ensuring the safety of both patients and healthcare professionals and the optimum utilization of online health-related interactions.

## 1. Introduction

Digital and information technologies have transformed the accessibility and quality of healthcare services. Globally, Electronic Health (E-health) has been introduced into the healthcare system by over 85% of the World Health Organization (WHO) member countries, with 50% having an established E-health strategy and 78% applying social media (SM) in the promotion of healthcare messages and services [1]. Concerning the Arab world, the Gulf Cooperation Council (GCC) countries have witnessed significant expenditures and development in recent years in telecommunications and E-health infrastructure [2]. The greatest effect of telehealth has been observed when applied for communication or monitoring of chronic illnesses, with improvements in fatality rates, life quality, and hospitalisations [3]. During the COVID-19 pandemic, the use of telehealth and telemedicine increased greatly and played an immensely positive role in reducing its impact on a global scale [4–7].

The WHO defines telehealth, including telemedicine, as “the delivery of healthcare services, where patients and providers are separated by distance” [6]. E-health refers to the use of mobile devices, the internet, and telecommunications for medical or public health practices by medical professionals, the general population, and information technologists [1, 8, 9], while telemedicine indicates a direct physician-physician or physician-patient remote contact in a secured virtual environment for the provision of medical consultations [1, 8, 9].

Currently, the bulk of the scientific literature on telemedicine use focuses on topics such as its usability, cost-effectiveness [5, 10–12], platforms used, user characteristics [10, 13], application challenges and facilitators [14], and patient and physician satisfaction [15–17]. When dedicating the search to the ethical and legal aspects of telemedicine, these were not as thoroughly covered, with a very limited assessment of related real-life experiences of healthcare practitioners, their perspectives and attitudes, particularly in Arab countries [18, 19].

The Saudi Ministry of Health (MOH) has successfully launched several mobile applications recently, such as Mawid, Sehha, and Wasfaty, that facilitated online booking of medical appointments, provision of virtual medical consultations, and dispensing of medications through an electronic prescription at times suitable for the patient [11]. Likewise, health promotion messages are regularly distributed by the MOH affiliated accounts on SM platforms [20]. Nonetheless, most of these services operate at a primary healthcare level. The provision of telemedicine for advanced healthcare services, on a large scale, is yet to be realized. Furthermore, in responding to health inquiries from the general community and patients, the healthcare practitioners' personal use of smart phones and SM platforms is common practice locally. This practice is neither well defined nor well researched, creating a grey area, a “transformation gap”, that doctors must venture in to cope with the direction of innovation necessary nowadays [21].

The success of implementing and maintaining telemedicine in the healthcare system, and on a national level, depends on the competency and efficiency of medical professionals to utilize such technology [22]. Research has shown that improved physicians' knowledge and understanding of telemedicine and its legal aspects significantly influence their attitude toward using it [23]. Thus, there is an urgent need to assess physicians' experience and knowledge regarding telemedicine implementation and its governing regulations in Saudi Arabia (KSA). Such information is essential to expand the national database on these topics and inform both junior and senior physicians' education and training programs.

The current paper examines Saudi physicians' perception of the use of telemedicine in patient care, their awareness of guidelines for health-related use of telemedicine, and their knowledge, attitude, and practice related to the ethical and legal regulations governing the use of telemedicine in the healthcare context.

## 2. Materials and Methods

This was an observational cross-sectional study conducted among physicians working at the College of Medicine, Imam Abdulrahman Bin Faisal University (IAU), and its affiliated academic tertiary hospital, King Fahad Hospital of the University (KFHU), Eastern province-KSA. Both IAU and KFHU were chosen to represent a typical, large university in KSA (IAU), and KFHU is a typical large tertiary academic hospital with a 440 beds capacity serving different groups of patients and providing 3 main types of services: curative services, teaching/training services and research.

The study participants were selected according to the following inclusion and exclusion criteria. The inclusion criteria were: physicians, males and females, working at any of the different departments/units of the College of Medicine/IAU and its affiliated KFHU, and who were involved in the teaching and training of under- and postgraduate students in the basic or clinical medical sciences. The main exclusion criteria were as follows: physicians working outside IAU or KFHU, physicians not involved in teaching/training of students, or not completing the study questionnaire, physicians in training (interns or residents), and other health care worker categories (nurses, pharmacists, laboratory personnel, etc.).

All eligible physicians available during the period of data collection were invited to participate, with the aim of full coverage of all physicians in the basic and clinical subspecialties of the College of Medicine/IAU and KFHU. Data were collected between August 2019 and March 2020 via a structured, pretested, self-administered questionnaire. The questionnaire was distributed to 220 eligible physicians at the College of Medicine and KFHU (source of eligible physicians' number: IAU human resources office), via face-to-face meeting with the physicians themselves or their respective unit's secretary. A final sample of 101 physicians completed our survey, approximately a 50% response rate. Reasons for nonparticipation were either “not having time”, “not interested,” being on leave at time of data collection, or providing incomplete responses.

The study questionnaire examined physicians' perception about the use of telemedicine [social media (SM) and medical applications (apps)] in patient care, their awareness of guidelines for health-related use of telemedicine, and their knowledge, attitude, and practice related to the ethical and legal regulations governing the use of SM and medical apps in the healthcare context. The use of telemedicine and awareness of guidelines were assessed using multiple-choice format questions (Yes/No), while the knowledge, attitude, and practice related to the ethical and legal regulations were evaluated with a 5-point Likert scale (strongly agree, agree, not sure, disagree, and strongly disagree).

The study questionnaire was constructed, by the research team, after review of the relevant literature on telemedicine related ethical and legal guidelines. It was then evaluated for content validity and comprehensiveness by independent physicians experienced on the topics investigated. The questionnaire was then piloted on another independent sample of 5 physicians to assess the feasibility of the study process. Feedback from the pilot about the understandability, acceptability, and length of the questionnaire was used to improve the survey questions. Additionally, Cronbach's Alpha estimate was 0.72 indicating good internal consistency for the 16 main items tested in our questionnaire (excluding demographic variables). Pilot study data were excluded from the final analyses presented in this article.

Efforts to prevent common method bias (CMB) and variance, at the design and implementation stages, included the following:

1. The wording of questions was made clear, concise, and accurate, and pretesting (above) was used to further improve and refine item wording
2. To limit extreme responses and (non)conformity-biased response styles, wording of questionnaire items was interchanged between different formats including positive, action, neutral, or questioning statements
3. To avoid that respondents regard any similarity in our multi-item scale measurements as repetitive, the wording of scale items was presented in diverse formats (above) and different scale response options were used (yes/no, and a 5-point Likert scale options). This is expected to reduce the chance of respondents using their response to one item as guide to answer related question(s)
4. Prior to data collection, all participants were assured that all responses would be handled anonymously, only accessible to the research team and used solely for research purposes. This is expected to encourage respondents to be open and honest when answering the questionnaire items

Furthermore, testing for common method bias was done. Harman's single factor test was performed, via exploratory factor analysis, in SPSS [the statistical package for social sciences IBM SPSS Statistics for Windows, version 28 (IBM Corp., Armonk, N.Y., USA)]. The total variance explained by a single factor is 16.693% which is less than 50% suggesting that CMB does not affect our data, hence our results.

Collected data were coded and analysed using SPSS. The results were organized and presented in tables and figures as frequencies, percentages, and summary statistics.

In addition, a checklist benchmarking procedure similar to other published studies [24, 25], is used to evaluate and compare our study results with the relevant key studies in the field. In such a process, it is important to provide checklist benchmarking that contains comparison points for evaluation of the currently presented results according to various characteristics. The comparison points reflect issues that must be measured and addressed in physicians' training on, and utilization of, telemedicine in health care. Checklist benchmarking is practical and important to measure how the presented analyses and results contribute alongside key works. The comparisons are based on whether the compared works covered the issues addressed in the listed comparison points. Checklist benchmarking comparisons are also presented. Ethical approval for this study was obtained from the Institutional Review Board (IRB) of Imam Abdulrahman Bin Faisal University, Saudi Arabia. Written Informed consent was obtained from all study participants prior to data collection, and they were assured that all data will be kept confidential and that they had the right to withdraw from the study at any time.

## 3. Results

Table 1 depicts the demographic characteristics of the participants. Of the respondents, 62 (61.4%) were males, 46 (45.5%) were in the age group of 35 to 44 years, and 62 (61.3%) were Saudi. The majority were from clinical sciences specialties corresponding to 85 (84%), and 53 (52.4%) had more than 10-year experience in undergraduate teaching.

**Figure 1:**
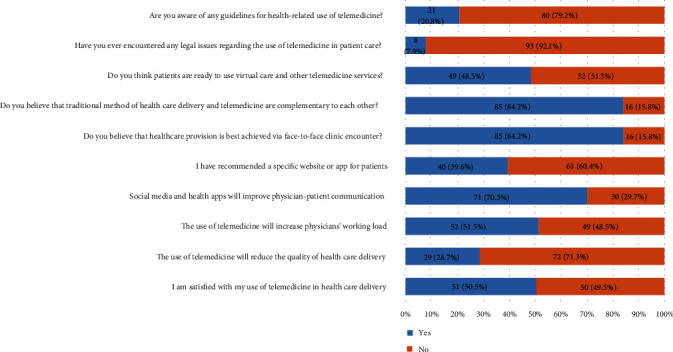
Use of telemedicine (social media and health apps) in patient care.

Among the participating physicians, 58 (57.4%) revealed using smart devices to deliver patient care. As seen in Figure 1, 21 (20.8%) of the respondents reported familiarity with telemedicine guidelines regulating its use for healthcare purposes, and 8 (7.9%) of the respondents have encountered legal issues in the use of telemedicine for patient care. Although 52 (51.5%) of the surveyed population were doubtful about patients' readiness for virtual care, 85 (84.2%) of them believed telemedicine to be complementary to the traditional method of healthcare delivery, and 85 (84.2%) of physicians considered face-to-face clinic encounters to be the best method for healthcare provision. Recommending a specific website or app for patients was done by 40 (39.6%) of the physicians, and 71 (70.3%) of them believed that SM and health apps will improve physician-patient communication. Another 52 (51.5%) of respondents consider the use of telemedicine to increase physicians' workload, and 72 (71.3%) disagreed that telemedicine will reduce the quality of health care delivery, with 51 (50.5%) being satisfied with their use of telemedicine in health care delivery.

**Figure 2:**
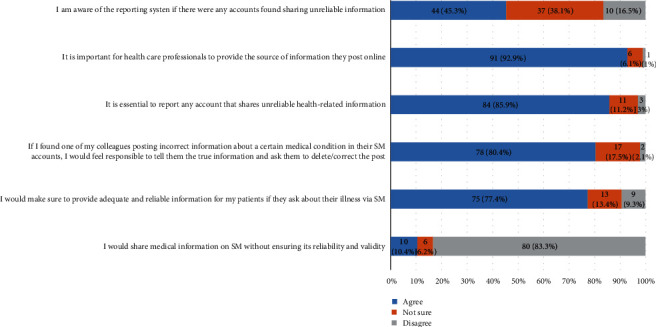
Ethical aspects regarding the reliability of information (social media (SM) and health apps).

Figure 2 demonstrates physicians' awareness about ethical aspects of the use of SM and medical apps in patient care, where 44 (45.3%) physicians were aware of the reporting system if they found any accounts sharing unreliable information and the rest were either unsure (37 (38.1%)) or unawareof such reporting systems (10 (16.5%)). Nonetheless, 84 (85.9%) of respondents agreed that it is essential for healthcare providers to report any account that shares unreliable health-related information, with 11 (11.2%) being unsure of this. Moreover, 78 (80.4%) of the participants would inform and correct their medical colleagues if they found them posting incorrect information, in contrast to the 17 (17.5%) that were uncertain about following the same action. Almost all 91 (92.9%) respondents agree it is important for healthcare professionals to provide the source of information they post online, and 80 (83.3%) would not share medical information on SM without ensuring its reliability and validity. Furthermore, 75 (77.4%) of our surveyed physicians would provide adequate and reliable information for their patients if they ask about their illness via SM, with 13 (13.4%) unsure and 9 (9.3%) disagreeing to do so.

**Figure 3:**
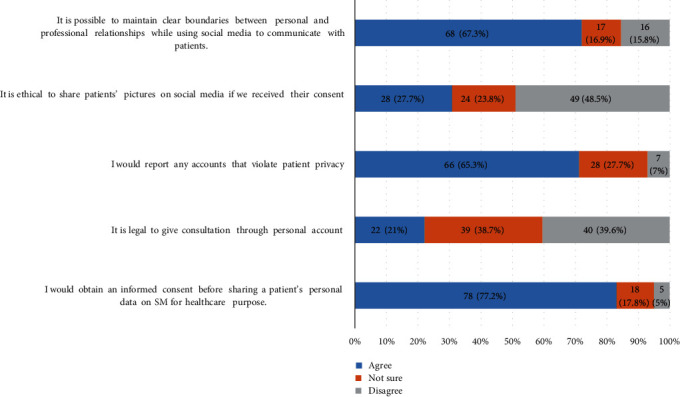
Patient privacy and confidentiality on social media (SM).

As illustrated in Figure 3, 68 (67.3%) of the participants agreed that it is possible to maintain professional boundaries with patients while using SM. Regarding sharing patients' pictures on SM with their consent, 28 (27.7%) of the respondents perceived this act to be ethical, 49 (48.5%) of them disagreed, and 24 (23.8%) were unsure. Moreover, 66 (65.3%) would report any account violating patients' privacy, while 28 (27.7%) were unsure and 7 (7%) disagreed. Regarding obtaining an informed consent before sharing patients' personal data on SM for health care purposes, 78 (77.2%) agreed they would do this, 18 (17.8%) were unsure, and 5 (5%) disagreed. Furthermore, 39 (38.7%) of the professionals were unsure whether it was legal to give consultation through personal accounts, 40 (39.6%) disagreed, and 22 (21%) agreed.

**Figure 4:**
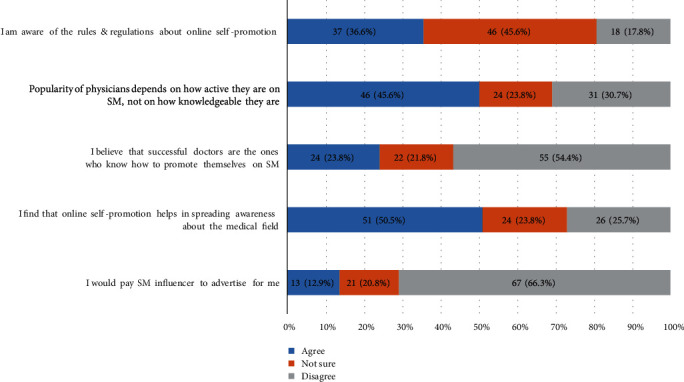
Self-promotion on social media (SM) and online platforms.

Figure 4 conveys the perception of the participants towards self-promotion on online platforms. Concerning physicians' awareness of the rules and regulations of online self-promotion, 46 (45.6%) of them were unsure and 18 (17.8%) disagreed, while 37 (36.6%) confirmed their awareness. Of the respondents, 46 (45.6%) agreed that physicians' popularity on SM depends on their activity level on these platforms rather than their clinical knowledge with the remaining either unsure (24 (23.8%)) or in disagreement with this (31 (30.7%)). Moreover, 55 (54.4%) of them did not agree that successful doctors are those who know how to promote themselves on SM while 24 (23.8%) believed it. The results indicate that 51 (50.5%) of the participants believe that online self-promotion helps raise public awareness of the medical field; however, 24 (23.8%) and 26 (25.7%) were unsure of and disagreed with this claim, respectively. What is more, 67 (66.3%) of our physicians would not pay SM influencers to advertise for them; nonetheless, 21 (20.8%) were unsure and another 13 (12.9%) would pay such an influencer.

Furthermore, the findings of this study are assessed through a checklist benchmarking procedure, where key aspects of our results are compared with important benchmark studies in the field (Table 2). The key points compared and illustrated in this comparison include the following: (1) physicians' familiarity with telemedicine guidelines and laws, (2) physicians' encountering legal issues while using telemedicine modalities in patient care, (3) maintenance of professional boundaries between physician and patients when interacting on SM, (4) correcting and/or reporting colleagues who happen to post incorrect online health-related information, (5) physicians' online self-promotion activities and regulating guidelines, and (6) online self-promotion and physicians' paying SM influencers to promote themselves or their institutions. Table 2 demonstrates whether or not the benchmark studies cover the comparison points addressed. The first three benchmark studies covered one comparison point each [6, 22, 26], while the fourth benchmark study covered 2 points [19]. Although the study conducted by Atiyeh et al. in 2020 was able to score 50%, it did not cover various other physician (online) practice related aspects such as number of physicians that encountered legal issues, the number of physicians that are willing to correct or report unreliable information posted online, and whether doctors are willing to pay a SM influencer for advertisement of their practices [27]. On the other hand, the current study addresses all compared important telemedicine use related aspects and adds valuable practical insight to prevalent knowledge on related physicians' training and awareness programs.

## 4. Discussion

Our study sample covers physicians, from both the basic and clinical medical specialties, with more than 10 years of experience in clinical care and training of under- and postgraduate trainees. We report that 57.4% of our participants use smart devices in healthcare delivery but only 20.8% of them know of telemedicine guiding regulations. Due to the rapid emergence and evolution of digital technology in the healthcare sector, the need for healthcare practitioners to be well trained and prepared for the use of telemedicine has become ever more pressing.

Our finding (57.4% of physicians using smart phones in the delivery of patient care) is expected and in line with before and after COVID 19 telemedicine use rates (33%-72%) reported among physicians in KSA [3, 4]. The complexity of care provided by large healthcare institutions, with an inconsistent provision of telemedicine training between these institutions [22], might have contributed to this variability in the telemedicine adoption rate. The use of telemedicine modalities, SM, and mobile applications in healthcare has specific ethical and legal aspects that require targeted physician training [19].

In connection to the above use rate, only 20.8% of our respondents were aware of any guidelines regulating the use of telemedicine for healthcare purposes; an expected result comparable to the 33.2% knowledge frequency of guidelines previously reported among Saudi physicians [22]. Notably, in the present study, reduced awareness of guidelines did not necessarily decrease the use of smart phones for patient care by our participants. Although there are no clear guidelines for the use of instant messaging applications globally [28], they are one of the most commonly used telemedicine modalities in KSA [26]. Telemedicine regulations in the country were only recently discussed by the National (Saudi) Health Information Centre in 2018 [29]. Internationally, the American Medical Association (AMA) and the American Telemedicine Association (ATA) have developed codes and guidelines for the use of telemedicine; these can serve as useful resources for physicians as well as related training and regulating bodies [30, 31].

Our data further reveal that 8% of our physicians have encountered legal issues with telemedicine. Although Saudi physicians were found by previous studies to be concerned about the legal aspects of telemedicine use [6, 22, 26], to our knowledge, ours is the first study to report an estimate of actual legal instances among KSA physicians, at a surprisingly high rate of approximately 1 in 13 physicians who use telemedicine in patient care. Uniform institutional adoption of telemedicine regulating guidelines, awareness of healthcare professionals and the public of these legal aspects, and the presence of clear regulating authorities are all necessary for the protection of all parties involved when utilizing telemedicine in healthcare delivery.

Patients' cultural beliefs and lack of awareness are among the barriers to wide telemedicine implementation reported in KSA and globally [32, 33]. Our results reflected this, where 51.5% of respondents thought patients were not ready to use virtual care and telemedicine services. Furthermore, 84.2% of our sample believed that face-to-face clinic encounters with patients were the best when delivering healthcare. This is an expected result and echoes national and international reports where 53% of Saudi doctors [26] and 60.8% of Australian physicians [34] were uncomfortable using online means to communicate with their patients. The inability to perform a full physical examination of patients, as reported by Saudi physicians [7], might be one of the reasons for physicians' preference for clinic-based, in-person visits.

Nevertheless, the majority (84.2%) of physicians in our study considered telemedicine to be complementary to traditional healthcare, with 70.3% believing telemedicine can improve physician-patient communication and another 39.6% had recommended a website or mobile app to their patients. Both E-health and SM reportedly offer patients easy access to health-related information [7, 21, 26, 35–37]. The perception that telemedicine will not reduce the quality of healthcare delivery, highlighted by our data (71% of participants), is in concordance with the results of other national studies where studied physicians viewed telemedicine to increase the quality of healthcare [7, 35].

Telemedicine is found to increase work-related stress among physicians in KSA; interestingly, it also increased their work satisfaction [35]. Our findings also reflected this, where about half (51.5%) of the participants believed telemedicine would increase physicians' workload, with an equal proportion of physicians (50.5%) satisfied with their use of telemedicine in healthcare delivery. These are expected results and might be due to physicians perceiving the related necessary additional training, administrative tasks, and safeguarding against any technical errors that may arise, as increased workload.

Overall, our physicians displayed a good knowledge of the ethical codes, as designated by the AMA [38], regarding the use of SM in healthcare including the need for providing the source of information when posting online (92.9%), supporting the provision of adequate and reliable information to online consultations through SM (77.4%), and discouraging the behaviour of sharing medical information without ensuring its reliability and validity (83.3%). This result is not surprising as these conduct codes resemble those regulating traditional, “bed-side,” medical care familiar to physicians. What is more, 85.9% of respondents agree, as anticipated, with the ethical responsibility of physicians to report online accounts sharing unreliable or incorrect health information. This is comparable to the findings of a previous study reporting that 70% of physicians in KSA felt obliged to correct any inaccurate information posted online [26]. The AMA considers physicians responsible for reporting unprofessional online content posted by their colleagues to the appropriate authorities [38]. Nonetheless, 38.1% of our respondents were unsure and 16.5% lacked awareness of relevant reporting systems. Unfortunately, if there is no clear definition or awareness of such authority, physicians might report these incidents to the regulating bodies of the SM platforms or applications in question, but in this case, effective action against this phenomenon might not be taken. Efforts should be made to establish more of such regulatory authorities, both nationally and internationally, and increase awareness of relevant proper guidelines and procedures among both physicians and healthcare consumers [18, 19].

Approximately, a third (32.7%) of our participants are unsure or disagree that it is possible to maintain clear professional boundaries with patients on SM. However, surprisingly, the majority (67.3%) of our physicians are optimistic about their ability to maintain such boundaries when interacting with patients on SM. This is a serious issue, as maintenance of boundaries on SM can be challenging and may negatively affect physicians' job opportunities and acceptance in residency programs [39, 40]. Furthermore, from a regulatory ethical aspect, the code of medical ethics by the AMA on the use of SM even suggests separating personal and professional accounts on online platforms to help maintain professional boundaries [38].

Concerning patient confidentiality on online media, our study physicians perceived imaged and nonimaged patient data differently. As anticipated, almost half (48.5%) opposed sharing a patient's picture on SM even if de-identified and patient informed consent was obtained. Of note here, according to the legal and court interpretations of the American Health Insurance Portability and Accountability Act (HIPAA), there are no legal implications for sharing patients' radiological images without their consent if they were deidentified [41]. On the other hand, over 77% of respondents would share a patient's data on SM if it were to be used for healthcare purposes and patient informed consent was secured. This seemingly new behaviour trend among physicians is likely facilitated by the ease and speed of data communication offered by online routes, as compared to the more traditional face-to-face and hard-copy-written communication methods [42]. In a study published in 2020 that is aimed at measuring the amount of identifiable information that healthcare professionals would share about their patients on Twitter, physicians were more likely to disclose patient identifiable information than other medical professionals [43]. Such violations of patient confidentiality will no doubt be faced with complaints that could lead to detrimental legal effects on a physician's career as seen in investigations undertaken by the General Medical Council (GMC) in the United Kingdom [44].

More than two-thirds of our study respondents were either unsure or did not think giving consultations through personal accounts was legal (38.7% and 39.6%, respectively). This is an expected and satisfactory result, demonstrating physicians' understanding of the importance of providing online care through appropriate, regulated, and encrypted channels [27, 41]. If deemed unavoidable, a physician is urged to give generalized standard responses when answering patients' inquiries through SM, as mentioned in previous reviews [41].

We report an unexpectedly high percentage (45.6%) of uncertainty among physicians regarding the rules and regulations of online self-promotion. This is probably a consequence of the above low rate of awareness among our sample of telemedicine regulating guidelines. This finding is concerning considering SM platforms have become a major marketing tool and a means to increase physicians' recognition by the public. This is evidenced by the role online promotion which has been shown to play, both in KSA and, internationally, in connection with specific health services such as cosmetic surgery [27, 45–47]. Patients, nowadays, are increasingly reliant on SM platforms to obtain health information and interact with physicians [27, 45–47]. Therefore, as its use by both physicians and patients is unavoidable, there is an urgent need to raise awareness among both parties of the rules and regulations regulating online health-related media content and interactions. Moreover, almost half of our respondents believed that a physician's popularity on SM depends on their account activity rather than their knowledge. Another worrying result, as previous review articles indicated, this might shift physicians' focus from patient care to becoming more popular online [27, 45–48]. Nevertheless, and as expected, the majority (54.4%) of our participants did not attribute a physician's success to his/her ability to promote themselves on SM. This is also confirmed in the literature, as some research on the use of SM in marketing found that the amount of engagement SM influencers' gain does not necessarily affect consumers' attitudes [47].

Further on the issue of online self-promotion, 51% of our study population perceived online self-promotion as a potentially useful tool in increasing the public's awareness regarding the medical field. This would assist consumers in better navigating the ever-increasing complexity of healthcare services. The published literature, in fields other than healthcare, has also shown that such online advertisement strategy has increased in recent years, as SM influencers are usually friendly and the public might regard them as trustworthy sources of information on different products and services [47]. However, previous reports on the issue concluded that certain physician groups, surgeons for instance, were more likely to post self-promotional than educational content on SM and were more likely to perceive SM as a means to gain more patients and profits [27]. Nevertheless, as anticipated, the majority (66.3%) of our physicians reject the notion of paying SM influencers to advertise for themselves.

A key strength of this study is that we conducted it in one of the larger teaching hospitals in KSA. Thus, the results are more likely to represent the leading institutions in the country in terms of telemedicine implementation by junior and senior physicians. Moreover, our sample included physicians who have experience in the clinical teaching and training setting, with about two-thirds having more than 5 years of experience and 50% even more than 10 years of relevant experience. Nonetheless, the findings of this study have to be considered in light of some limitations. Our sample size of 101 physicians might be considered relatively small; nevertheless, it covered physicians from both the basic and clinical specialties in proportions similar to those in comparable institutions in KSA. In addition, we think our results highlighted essential aspects regarding the utilization of telemedicine in healthcare.

## 5. Conclusion

The use of telemedicine modalities, SM and mobile applications, in healthcare has specific ethical and legal aspects that require targeted physician training. We report that more than half of our participants use smart devices in healthcare delivery but only 20.8% of them know of telemedicine guiding regulations. Our data further reveal that 1 in 13 physicians has encountered legal issues with telemedicine; representing the first report of an estimate of actual legal instances among KSA physicians, while more than two-thirds of our study respondents were either unsure or did not think giving consultations through personal accounts was legal. Furthermore, almost half of our respondents were uncertain of the rules and regulations governing physicians' online self-promotion. This is a vital finding considering the increasing presence of physicians on SM platforms and the reliance of patients on these platforms as health information sources and for interaction with physicians. Future research addressing the experience of other healthcare provider categories, and in differing contexts, is warranted. Due to the rapid emergence and evolution of digital technology in the healthcare sector, the need for healthcare practitioners to be well trained and prepared for optimal application and utilization of telemedicine has become ever more pressing. The presented findings have important current and future implications for improving clinical practice, as well as fine-tuning and defining desired training outcomes in present-day continuous professional development programs.

## Figures and Tables

**Table 1:** Characteristics of the study population.

Characteristic	Number	Percent
*Age (years)*		
25-34	15	14.9
35-44	46	45.5
45-54	21	20.8
55-64	18	17.8
≥65	1	0.9
*Gender*		
Male	62	61.4
Female	39	38.6
*Specialty*		
Basic sciences	16	15.8
Clinical sciences	85	84.2
*Experience(years) in undergraduates teaching*		
<1 year	1	1
1–5 years	24	23.8
5–10 years	23	22.8
>10 years	53	52.5
*Total*	*101*	*100*

**Table 2:** Comparison points between benchmark articles and the current study.

	Comparison points	Benchmark#1 (Albarrak et al. 2019)	Benchmark#2 (Alanzi et al. [26])	Benchmark#3 (Kaliyadan et al. [6])	Benchmark#4 (Nittari et al. [19])	Benchmark#5 (Atiyeh et al. 2020)	Current study
1	Physicians' familiarity with telemedicine guidelines/laws	√	×	√	√	√	√
2	Encountering legal issues while using telemedicine modalities in patient care	×	×	×	×	×	√
3	Maintaining professional boundaries while interacting with patients on social media	×	×	×	√	√	√
4	Reporting or correcting colleagues posting incorrect online health-related information	×	√	×	×	×	√
5	Awareness of online self-promotion regulations	×	×	×	×	√	√
6	Willingness to pay a social media influencer for advertisement	×	×	×	×	×	√
Total score		16.67%	16.67%	16.67%	33.33%	50%	100%
Finding difference		83.33%	83.33%	83.33%	66.67%	50%	—

## Data Availability

The datasets used for the current study are available from the corresponding author upon reasonable request.
